# Methionine restriction and cancer treatment: a systems biology study of yeast to investigate the possible key players

**DOI:** 10.55730/1300-0152.2656

**Published:** 2023-05-23

**Authors:** Esra BÖRKLÜ

**Affiliations:** Koç University Hospital, Genetic Diseases Evaluation Center, Koç University, İstanbul, Turkey

**Keywords:** Methionine restriction, cancer, transcriptomics, systems biology, network analysis

## Abstract

**Background/aim:**

Dietary restriction, mainly carbon and/or methionine restriction are among the upcoming supporting interventions along with chemotherapy in various cancers. Although dietary restriction has been proven to be beneficial, the main cellular machineries affected by its administration lacks deeper information considerably, a notable pitfall in its use as a personalized nutritional approach.

**Materials and methods:**

In this study, cellular effects of methionine restriction on a yeast model are explored via systems biology approaches. The methionine biosynthesis network, constructed by integrating interaction data with gene ontology terms, was analysed topologically, and proved to be informative about the intertwined relationship of methionine biosynthesis and cancer. Experimentally, effects of methionine restriction on the yeast model were explored in vivo, with transcriptome analyses.

**Results:**

The integrative analysis of the transcriptional data together with the reconstructed network gave insight into cellular machineries such as TOR, MAPK, and sphingolipid-mediated signaling cascades as the mostly responsive cellular pathways in the methionine-restricted cases with Sch9p (functional orthologue of mammalian S6 kinase) being placed at the intersection of these signaling routes.

## 1. Introduction

A group of diseases which encompass abnormal cell growth, which has the potential to spread to other parts of the body are termed as “cancer”. Cancer is estimated to be the second leading cause of death globally in 2018, according to World Health Organization (WHO).

Since it is a disorder of abnormal growth, adopting starvation techniques in order to deprive tumors of energy were first proposed in the late 1920s by Otto Warburg, founder of the “Warburg effect” (Warburg, 1956). Otto Warburg’s study pointed to carbon, namely glucose starvation as a useful strategy. Later in the 1980s, methionine-addiction of cancers of all types (Mecham et al., 1983), probably for excess transmethylation reactions was discovered and named as “Hoffman effect” (Stern and Hoffman, 1984). It was later demonstrated that the proliferation and growth of several types of cancer cells were inhibited by methionine restriction (MR) (Cellarier et al., 2003). Moreover, MR has recently been shown to enhance the efficacy of chemotherapy and radiation therapy in animal models (Gao et al., 2019). This beneficial effect has been attributed, but not limited, to three machineries: reduction of oxidative stress (glutathione synthesis), regulation of transcription and translation (polyamine synthesis), and effects on DNA methylation (S-adenosyl methionine synthesis) (Wanders et al., 2020). Recent studies demonstrating the antiproliferative role of MR on colorectal (Zhou et al., 2022), triple negative breast (Jeon et al., 2016), and prostate (Lu et al., 2020) cancer cells further fortify the use of MR as a potential therapeutic agent.

*Saccharomyces cerevisiae*, the budding yeast, has long been used as a model organism for human cancers, both for the broader investigation of cellular machineries leading to cancer biogenesis (Cazzanelli et al., 2018) and for studying the response to anticancer agents (Matuo et al., 2012). Effects of MR have also been studied in yeast, although research was limited mainly to the field of yeast aging (Ruckenstuhl et al., 2014; Plummer and Johnson, 2019). The transcriptional and translational dynamics of *Saccharomyces cerevisiae* cells upon MR have also been explored via RNA-Seq and ribosome profiling recently (Zou et al., 2017), focusing mainly on the translational regulation by MR.

The current study is the first study where the integration of transcriptome and interactome data for yeast, by systems biology approaches, is conducted. The methionine biosynthesis network reconstructed in *S. cerevisiae* is used as a backbone to investigate potential alterations and crosstalks with other signaling networks when methionine is limited. The integrative analysis provides key proteins, processes, and/or pathways having a role in the demonstrated beneficial effect of MR on cancer treatment.

## 2. Materials and methods

### 2.1. Reconstruction of the methionine biosynthesis network

The methionine biosynthesis network was reconstructed via the Selective Permissibility Algorithm (SPA) (Arga et al., 2007; Börklü-Yücel and Ülgen, 2011). The 8 (HOM2p, HOM3p, MET6p, MET13p, STR3p, HOM6p, MET2p, MET22p) proteins which were manually curated to have the “methionine biosynthetic process” in their GO function term on Saccharomyces Genome Database (SGD) as of January 2023, were adopted as the seed proteins from which the network expands. The Annotation Collection table, the second input for the reconstruction algorithm, was created by pooling the process, function, and component GO annotations of the mentioned seed proteins only, downloaded from SGD, as of January 2023. The third input for SPA is the physical protein-protein interaction file obtained from BIOGRID, release 4.4.218. Briefly, the reconstructed network expands from these 8 seed proteins in such a way that a candidate protein is selected to be a member of the network based on its GO Terms and its physical interaction data: it is included in the network if and only if its three GO Terms are present in the Annotation Table and it interacts physically with one of the seed proteins. The included proteins constitute the “first neighbors” of the seed proteins, and the network continues to expand by taking these first neighbors as the new seed proteins for the rest of the automated algorithm until no new proteins are found to be added to the network.

### 2.2. Network analysis

For the topological analysis of the reconstructed network, the “NetworkAnalyzer” tool of Cytoscape software was used (Assenov et al., 2008). Highly densely connected proteins of the reconstructed network were identified with MCODE (Bader and Hogue, 2003) algorithm. Responsive subnetworks were created with “jactivemodules” plugin of Cytoscape, with the default settings (Ideker et al., 2002). Briefly, a z-score for a subnetwork A which has k members is calculated according to $z(\text{A})=\frac{1}{\sqrt{k}}\sum i\in A\;z_{i}$ where *z_i_* = *ϕ*^−1^(1−*p_i_*), with ϕ^−1^ being the inverse of the normal cumulative distribution function and p being the p-values of the nodes present in the subnetwork. The module score s(A) for the subnetwork A is the normalized value of this z-score with mean and standard deviation of scores z (A) over all k-node groups of the graph.

### 2.2. Experimental procedure

The strain used in the experiments was *ΔHO* derived from BY4742 background, *Matα; his3Δ1; leu2Δ0; lys2Δ0; ura3Δ0; YDL227c::kanMX4* obtained from EUROSCARF deletion collection. For the main cultures, overnight cultures grown in SDC media were diluted to an OD600 value of 0.1 and inoculated into fresh SDC media in micro-aerated flasks, with a working volume of 1:5. The cultures were then grown batch-wise at 30 °C and 180 rpm up to mid-exponential phase (OD600 ~ 0.6). At the mid-exponential phase, cells of the main culture were divided into three aliquots which were centrifuged at 6000 rpm for 5 min, washed twice with deionized, and distilled sterile water prior to their transfer in the treatment media. Treatment media comprised of fresh SDC (2% methionine) for the control case, SDC + 0.75% methionine for the methionine restricted case. Samples for transcriptome analyses were collected 2 h after the treatment. The experiments were done in biological triplicates.

### 2.4. Sampling and mRNA extraction

For mRNA extraction, 5 mL samples were collected, frozen in liquid nitrogen immediately, and stored at −80 °C prior to RNA extraction. RNA extraction was performed automatically with QiaCube using RNeasy Mini Kit (Quiagen), as described by the manufacturer.

### 2.5. Microarray data acquisition and processing

The qualitative and quantitative spectrophotometric analysis of RNA was done using UV-vis spectrophotometer (NanoDrop ND-1000, Thermo Fisher Scientific Inc., U.S.A). RNA integrity number (RIN) values were checked using a microfluidics-based platform (Bioanalyzer 2100 Agilent Technologies, USA) using RNA6000 Nanokit (Agilent Technologies, USA) and samples with RIN values 7–10 were processed. The microarray analysis steps comprising of the synthesis of cDNA, conversion of cDNA into a double-stranded DNA, transcription and synthesis of biotin-labeled aRNA from the double stranded DNA, purification and fragmentation of aRNA, and the final hybridization of aRNA were performed as described in the Affymetrix GeneChip^®^Expression Analysis Technical Manual.

Data were processed in R using raw cell files via the “affy” (Gautier et al., 2004) package of Bioconductor, with “rma” option chosen for background correction and “quantile” option for quantile normalization. Final expression values for the genes were obtained in log2 transformed values, differentially expressed genes were obtained by Student’s t-test GO Term enrichment results of the differentially expressed genes were obtained via web-based “gprofiler” server (Raudvere et al., 2019), with a Benjamini-Hochberg FDR value of 0.05 as the significance threshold. Data were submitted to ArrayExpress database, under the accession number E-MTAB-12860.

## 3. Results and discussion

### 3.1. The key proteins of the methionine biosynthesis network are linked to cancer

**Figure 1:**
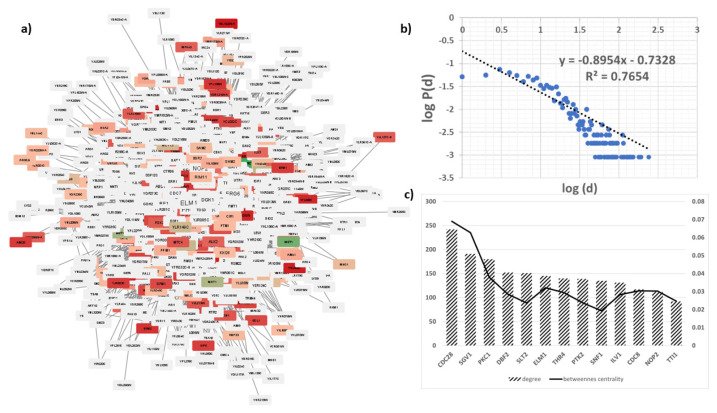
Analyses of the reconstructed network. a) Cytoscape view of the network, coloring based on the fold change (red and green colors represent upregulated and downregulated genes respectively); b) degree distribution plot of the network and c) key proteins of the network. The left axis represents degree values while the right axis is for BC values.

The methionine biosynthesis network, reconstructed with SPA, had the 8 genes which had the “methionine biosynthetic process” as their manually curated GO process term as the seed genes (Table). The annotation table used to expand the network, pooled terms of these seed proteins, comprised of 7 component GO terms together with 31 function and 29 process terms (Table). The final undirected network reconstructed had 1102 nodes and 10141 edges (Figure 1a). The topological analysis of the network revealed that it reflected the “small world” property of the biological networks, with network diameter and mean path length values of 5 and 2.7 respectively, despite the large node number of 1102. Moreover, the distribution of node degrees follows nearly a power law model (P(k) = k^−γ^) as in many other biological networks, with γ = 0.895 and an R^2^ value of 0.77 (Figure 1b). These parameters imply that the network in question contains numerous small, highly integrated modules as is the case for many other active biological networks.

The highly connected nodes, referred to as the “hubs” of the network, reflect the highly impactful genes in the network, around which various other system components (nodes) revolve. On the other hand, the nodes with high “betweenness centrality” (BC) values are the nodes through which shortest paths are traversed more heavily, i.e. they contribute more to the information flow in the network. With these descriptions in hand, the top 10 hubs and top 10 nodes with the highest BC values of the network are inspected closely (Figure 1c). A combination of these two lists resulted in 13 genes (*CDC28, SGV1, PKC1, DBF2, SLT2, ELM1, THR4, PTK2, SNF1, ILV1, CDC8, NOP2*, and *TTI1*) each with a human orthologue, known to be implied in various cancers (Figure S1).

Cdc28p (yeast orthologue of human CDK1) is a cyclin-dependent kinase controlling the mitotic cell cycle. Apart from the tight relationship between cell cycle abnormalities and senescence of cancer cells, CDK1 upregulation is known to be related to reduced survival time for colorectal cancer, liver cancer, and lung cancer (Kuang et al., 2019). Sgv1p, CDK9 in Homo sapiens, is another cyclin-dependent kinase, involved in the regulation of transcription elongation from RNA polymerase II promoters. Recent studies demonstrated that the inhibition of CDK9’s activity holds the potential to be a highly effective anticancer therapeutic (Mandal et al., 2021). Pkc1p, orthologue of human PKC, is a serine-threonine protein kinase proven to have important roles in carcinogenesis and can serve as a promising target for cancer treatment (He et al., 2022). STK38 is the human orthologue of another Ser/Thr kinase of the network, Dbf2p, which has a significant prognostic value in different cancers and is closely associated with cancer immunity (Liu et al., 2022). Slt2p is a member of the MAPK cascade in yeast, as its orthologue ERK5 in humans, and alterations of the MEK5/ERK5 signaling pathway have been described in several tumor cells (Monti et al., 2022). Members of another signaling pathway, mammalian AMPK signaling, are also among the key proteins: Snf1p and Elm1p. Snf1p (AMPK) along with its regulator Elm1p (CAMKK), is another promising potential target for cancer prevention and treatment (Li et al., 2015). Ptk2p, the yeast orthologue of human MARK2, is another protein kinase of the network. Overexpression of MARK2 in lung tumor cohorts is well established and novel roles in pancreatic cancers are also described for MARK2 (Zeng et al., 2022). Cdc8p, another key kinase, is the yeast orthologue of human DTYMK whose inhibition has been shown to restrain the growth of hepatocellular carcinoma and increase sensitivity to the therapeutic agent oxaliplatin (Sun et al., 2021). Nop2p is a methyltransferase whose human orthologue NOP2 has been demonstrated to be upregulated in colon cancer (Bi et al., 2019). Tti1p is a telomere length regulator, with the orthologue of TTI1 in humans. Overexpression of TTI1 in colon and lung cancer has recently been unraveled (Xu et al., 2021). The remaining two key proteins are directly related to threonine metabolism, Thr4p (THNSL2), and Ilv1p (SDS). Although a definite role for threonine metabolism in cancer has not yet been proposed, there are theories about fueling of the TCA cycle by threonine which results in the release of ATP for the required energy for oncogenic activities (Lieu et al., 2020).

The tight interconnection of these 13 hub proteins with various cancer types, has proven that the dynamics of the methionine biosynthetic process may indeed be one of the key factors in cancer biology.

### 3.2. Microarray analysis reveals that methionine restriction triggers sulfate assimilation while represses ribosome biogenesis pathways

**Figure 2:**
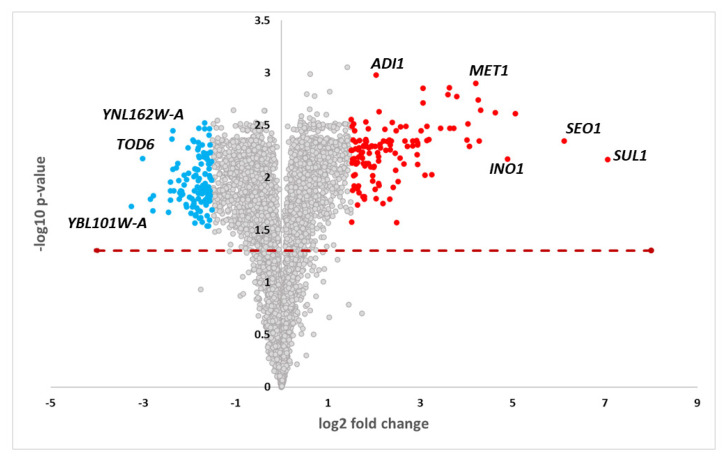
Volcano plot of the gene expression profiles. Red and blue denote the significantly up and downregulated genes, respectively.

Statistical analysis of the transcriptome data of methionine restriction vs. control case resulted in 247 differentially expressed genes (FDR p-value < 0.05, absolute fold change >1.5) of which 121 and 126 were down- and up-regulated in methionine restriction case, respectively (Figure 2, Table S1). The GO Term and KEGG pathway enrichment results of these differentially expressed genes gave two prominent results: Genes downregulated in methionine restriction case were primarily enriched with “Ribosome biogenesis” and “rRNA methylation” (p-val:2.54E-17) terms. This result means that in the dietary restricted case, ribosome synthesis and/or assembly was down-regulated probably due to the methylation defects of rRNA, since methionine is scarce. This fact may be one of the beneficial effects of methionine restriction on cancer cells since uncontrollably proliferating cancer cells need to produce ribosomes to sustain continuous proliferation and expand in numbers. In fact, recent work stressed the importance of targeting ribosome biogenesis as a potential therapeutic intervention in cancer (Zisi et al., 2022).

**Figure 3:**
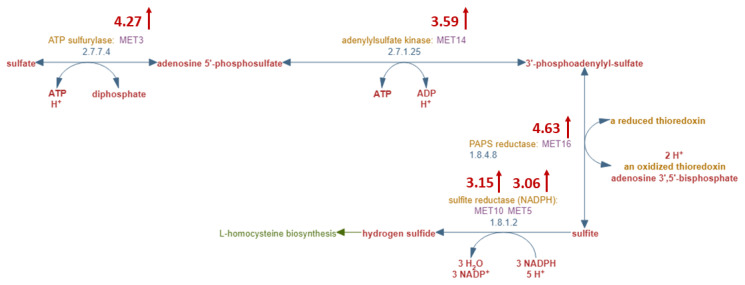
Sulfate assimilation pathway of yeast with fold change values in red (adapted from SGD).

The upregulated genes were enriched in “sulfate assimilation” (p-val:1.64E-11) pathway. When this pathway is closely scrutinized, it is seen that all the genes of the pathway are significantly upregulated in methionine restriction (Figure 3). The end product of this pathway, however, hydrogen sulfide, is a gasotransmitter and has been reported to have contradictory effects related to cancer biogenesis and progression. This controversial role of H_2_S in the cancer research field is explained by a bell-shaped (biphasic) model, in which lower concentrations of H_2_S display procancer effects while higher concentrations exhibit anticancer properties (Hellmich and Szabo, 2015). It is proposed in this study that methionine restriction increases endogenous H_2_S levels to a threshold level that exhibits anticancer properties. It is known that yeast cells grown in methionine restricted conditions prefer quiescence over senescence, and this fact occurs simultaneously with increased hydrogen sulfide production (Choi et al., 2019). To evade senescence with higher H_2_S production may support this level-altering hypothesis, although further experimentation is needed for a definite result.

Results of the microarray experiments are in line with the literature: according to Zou et al.’s study also, upregulated genes are enriched in the amino acid/methionine biosynthesis pathways whereas downregulated genes are enriched with terms pertinent to ribosomal processes (Zou et al., 2017). *MET1, MET3, MUP3, SUL1*, and *SUL2* are all among the commonly upregulated genes in both studies, genes involved in methionine biosynthesis. *ATG41* and *INO1* however, are among the significantly upregulated genes in this study only, hinting growth phase at which methionine is restricted may affect the transcriptional profiles.

Atg41p’s function is unknown but is known to be induced in autophagy-inducing conditions (Yao et al., 2015) and is required for autophagy and mitophagy. This result may point to *ATG41* for the lifespan-increasing role of MR via mitophagy (Plummer and Johnson, 2019). The tumor-suppressing role of mitophagy in the early stages of tumor development is also well-established (Ferro et al., 2020). Ino1p on the other hand, is involved in the synthesis of inositol phosphates and inositol-containing phospholipids, especially IP_6_, which in its turn enhances the anticancer effect of conventional chemotherapy, controls cancer metastases, and improves the quality of life in cancer patients (Vucenik et al., 2020). Thus, among other signaling pathways, another signaling pathway tightly intertwined with methionine restriction may be inositol-phosphate signaling machinery, according to the transcriptome analysis.

### 3.3. Integration of transcriptome data with methionine biosynthesis network points to Sch9p as a key regulator between MAPK, TOR, and sphingolipid signaling machineries in response to methionine restriction

To dynamically examine the reconstructed network in part 3.1, “jactivemodules” plugin of Cytoscape is adopted. Briefly, gene expression data of part 3.2 is integrated into the methionine biosynthesis network, yielding an active subnetwork which captures the dynamic information correlated with methionine restriction. Computational integration of the network and the transcriptomics profile leads to the extraction of context-dependent active modules, which mark regions of the network showing striking changes in molecular activity associated with methionine restriction.

**Figure 4:**
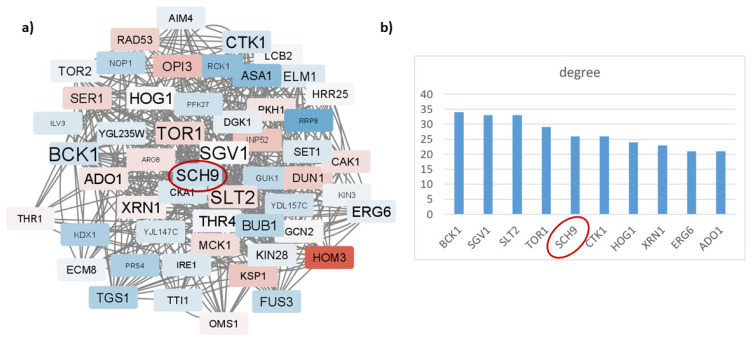
Results of the dense network. a) Cytoscape view of the dense network with label sizes depicting degree values and node colors based on expression values. Red and blue denote up and downregulation. b) Degree distribution of the nodes of the dense network. Sch9p is one of the hub proteins.

**Figure 5:**
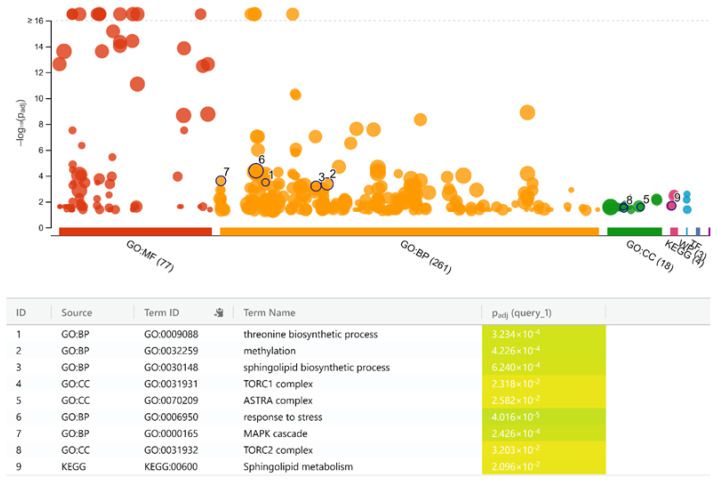
Enrichment results of the members of the dense network with the “gprofiler” web server.

The integration procedure resulted in a small subnetwork of 243 nodes and 1442 edges. This responsive subnetwork was then processed via MCODE algorithm to discover the densely connected components: only 3 modules with a score of >3 are found. These highly interconnected regions represent protein complexes and/or parts of a pathway which are triggered in methionine restricted cases. The combined analysis of these three modules yielded a single connected component, a dense responsive network of 53 nodes and 393 edges, referred to henceforth as the “dense network”. Enrichment results of this dense network gave insight into the cellular machineries affected in the methioninerestricted case. According to this analysis, methionine restriction affects TOR and MAPK signaling pathways along with sphingolipid biosynthetic process (Figures 4 and 5). A detailed, closer inspection of the members of this dense network provides a comprehensive interpretation of systems altered in methionine restriction.

Tor1p and Tor2p, orthologues of mammalian mTOR, are already expected to be one of the key nodes of the dense network since amino acid limitation has a direct impact on TOR signaling to regulate growth in response to nutrient stress in *Saccharomyces cerevisiae*. A similar phenomenon has also been described in *Homo sapiens* (Takahara et al., 2020). Fus3p, the yeast orthologue of human MAPK1 (ERK5), is another member of the dense network, and it was published that a hyperactive ERK5 has a direct role in tumorigenesis (Guo et al., 2020). Three enzymes of the network are playing a part in sphingolipid biosynthesis, thus sphingolipid-mediated signaling: Inp52p, Lcb2p, and Sch9p. Inp52p takes part in phosphatidylinositol (PI) production while Lcb2p catalyzes the formation of 3-dehydrosphinganine, the rate-limiting step in sphingolipid biosynthesis. Sphingolipids are bioactive molecules which have signaling properties and are recently been investigated for their potential roles in cancer and therapy (Li et al., 2022). Sch9p, the functional orthologue of mammalian S6 kinase, situates itself at the junction of these pathways (Figure 4): S6 kinase is phosphorylated by mTOR and ERK while it is inhibited by the sphingolipid signaling molecule ceramide (Wang et al., 2001; Magnuson et al., 2012; Jesko et al., 2019). It may thus be hypothesized that Sch9p integrates the information from TOR, MAPK, and sphingolipid-mediated signaling pathways in response to methionine restriction and subsequent beneficial antitumor effects.

## 4. Conclusion

Dietary restrictions emerged as new nutritional interventions which may enhance the efficacy of chemotherapeutic agents. Results of the current study revealed that the reconstructed methionine biosynthesis network has cancer-related orthologues as the hub nodes, fortifying the potential use of methionine restriction as a subsidiary intervention to fight cancer. Microarray analysis of cells subjected to methionine restriction pointed to hydrogen sulfide production as the triggered cellular machinery while ribosome synthesis was repressed. Both phenomena have been mentioned as therapeutic targets for various cancer types. Integration of transcriptome data with the reconstructed network broadened the perception of various signaling machineries such as TOR, MAPK, and sphingolipid-mediated pathways, each triggered in response to methionine restriction. Sch9p, the yeast orthologue of human S6K, seems to be the key player in the mentioned beneficial cellular response by taking place in the crosstalk between these different signaling routes. Further experiments which solely target S6K phosphorylation, or target S6K in combination with dietary restriction will provide valuable insights for antitumor therapy.

## Supplementary Information

Figure S1 The visualization of 13 hub genes and their first interacting partners in the network. Hub genes are in blue while the interaction partners are in pink.

## Figures and Tables

**Table:** List of seed proteins and their corresponding GO Terms, adopted as the Annotation Table.

Seed proteins		Annotation Table		
ORF	GENE	Compartment	Function	Process
YDR158W	HOM2	GO:0005575	GO:0000166	GO:0000096
YER052C	HOM3	GO:0005634	GO:0003674	GO:0000103
YER091C	MET6	GO:0005737	GO:0003824	GO:0006520
YGL125W	MET13	GO:0005739	GO:0003871	GO:0006555
YGL184C	STR3	GO:0005777	GO:0004072	GO:0006790
YJR139C	HOM6	GO:0005829	GO:0004073	GO:0008150
YNL277W	MET2	GO:0005886	GO:0004121	GO:0008652
YOL064C	MET22		GO:0004412	GO:0009058
			GO:0004414	GO:0009067
			GO:0004489	GO:0009082
			GO:0005524	GO:0009085
			GO:0008168	GO:0009086
			GO:0008172	GO:0009088
			GO:0008270	GO:0009089
			GO:0008441	GO:0009090
			GO:0016301	GO:0009092
			GO:0016491	GO:0009097
			GO:0016620	GO:0016053
			GO:0016740	GO:0016078
			GO:0016746	GO:0016310
			GO:0016747	GO:0019346
			GO:0016787	GO:0032259
			GO:0016829	GO:0035999
			GO:0016846	GO:0042538
			GO:0030170	GO:0046854
			GO:0046872	GO:0046855
			GO:0046983	GO:0071266
			GO:0047804	GO:1901566
			GO:0050661	GO:1901605
			GO:0051287	
			GO:0071949	
