# Supplementary figures and images for: Methionine restriction and cancer treatment: a systems biology study of yeast to investigate the possible key players
Source: Turk J Biol. 2023 May 23;47(3):208–17. doi: 10.55730/1300-0152.2656 (PMC10388026; doi:10.55730/1300-0152.2656)

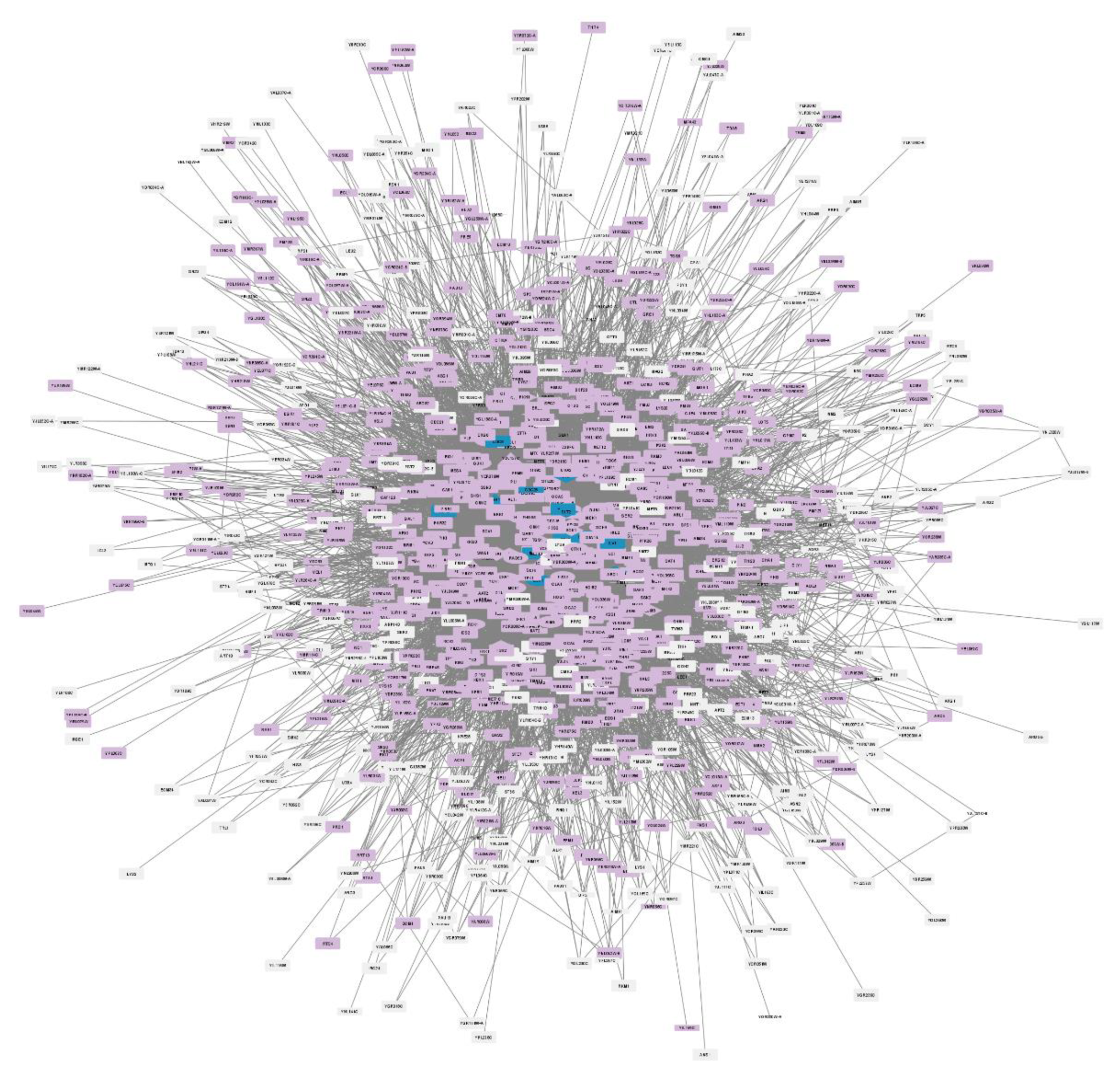

Supplement: Figure S1 — The visualization of 13 hub genes and their first interacting partners in the network. Hub genes are in blue while the interaction partners are in pink. [file turkjbiol-47-3-208s1.tif]
